# Supplementary material for: Anisotropic diffuse growth in Arabidopsis thaliana stigma papillae
Source: Plant Reprod. 2025 Jun 4;38(2):13. doi: 10.1007/s00497-025-00523-3 (PMC12137461; doi:10.1007/s00497-025-00523-3)
Supplement: Supplementary file 1 — Supplementary file1 (PDF 1513 kb) [file 497_2025_523_MOESM1_ESM.pdf]

Supplemental Table 1: Regression Data from Col-0 and Diffuse Growth Mutant Growth Curves
(y=stigma papillae length; x=stigma papillae width)

	**Col-0**	***any1***	***ktn***	***fass***
Exponential Fit	$y=1.6191e^{0.2241x}$	$y=3.8971e^{0.1256x}$	$y=3.8075e^{0.1212x}$	$y=4.4088e^{0.00951x}$
Linear Fit of ln-Transformed Data	y=2.6402x-3.2488	y=1.9956x-1.956	y=1.8784x-1.6911	y=1.541x-1.0537
R^2	0.9086	0.9665	0.9646	0.9685
**Anisotropic Growth Factor (AGF)**	**2.6402**	**1.9956**	**1.8784**	**1.541**
AGF Standard Deviation	0.0054	0.0028	0.0019	0.0022
Number of Flowers (n)	112	91	103	67
Significance of AGF Compared to Col-0	NA	9.4374E-195	5.7127E-171	7.8248E-203

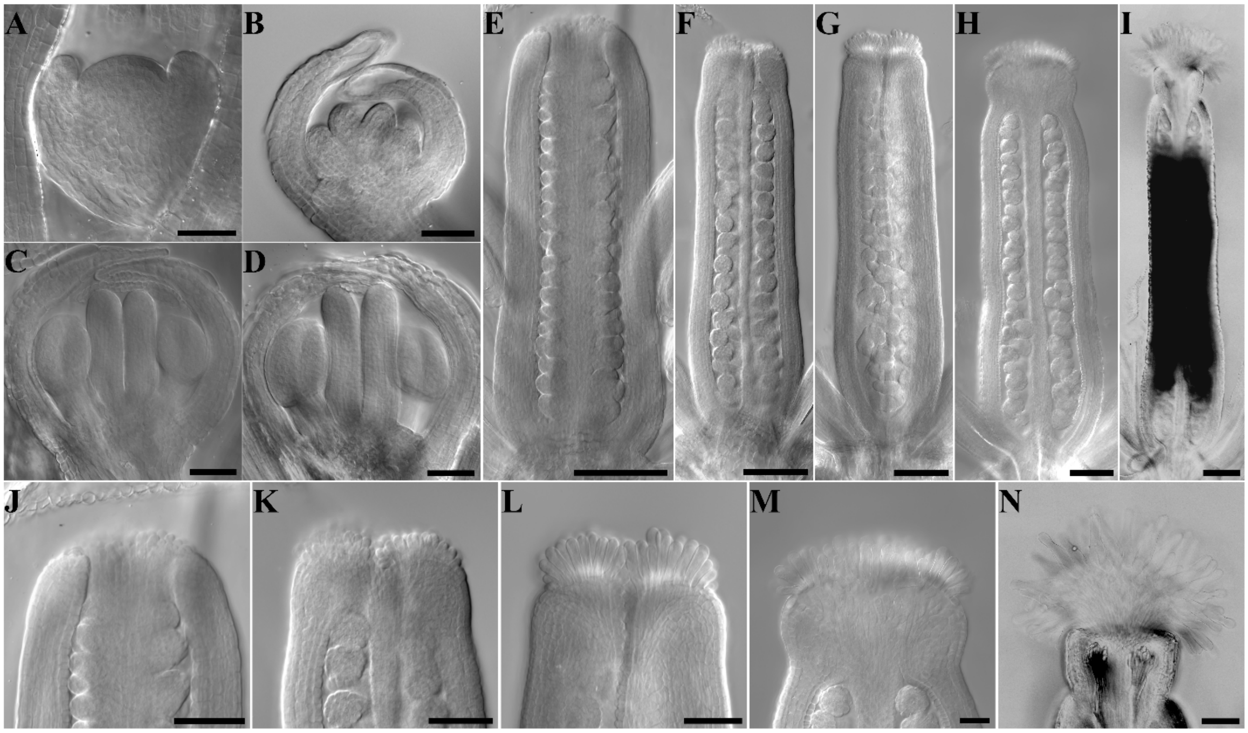

Supplemental Figure 1: Gynoecium and Stigma Development Over Floral Stages

(A-I) DIC images of fixed and cleared gynoecia representatives from various floral stages: Stage 3 (A), Stage 6 (B), Stage 7 (C), Stage 8 (D), Stage 9 (E), Stage 10 (F), Stage 11 (G), Stage 12 (H), Stage 13 (I). (J-N) closeups of stigma from (E-I). Scale bars: 50 µm (A-D, J-K), 100 µm (E-I).
